# The Causation of Cancer

**Published:** 1924-12

**Authors:** J. Ellis Barker

**Affiliations:** Albion Lodge, Fortis Green, East Finchley, N. 2


### CORRESPONDENCE.

[Correspondence on all subjects is invited, but we cannot in any way be responsible for the opinions expressed by our correspondents. No communication can be entertained if the name and address of the correspondent are not given as a guarantee of good faith, but not necessarily for publication. All correspondents should write on one side of the paper only, and we cannot find space for very long letters.]

### The Causation of Cancer.

*To the Editor of* The Hospital and Health Review.

Sir,—The report of the Board of Control (Lunacy and Mental Deficiency) just published gives on page 34 most interesting and important figures, comparing the cancer death-rate among the people in general and the cancer death-rate in mental institutions. Among the people in general, the cancer death-rate is 12.4 per cent. and among the inmates of mental institutions it is only 3 per cent. In other words, cancer is more than four times as deadly outside than inside mental institutions.

In my recently published book, "Cancer, How It is Caused, How It Can Be Prevented," I have endeavoured to show by an overwhelming mass of facts that cancer is caused by chronic poisoning and vitamine starvation, that it is largely due to dietetic mistakes, and especially to the consumption of concentrated, manipulated, de-vitaminised and demineralised foodstuffs, that it is a disease of civilisation and of over-civilisation. The extraordinary figures supplied in the report mentioned strikingly confirm my views. The inmates of the mental institutions, who number about 100,000, are rather poorly and plainly fed. Their diet is the diet of fifty years ago. They do not get the "scientific" foods which are now so generally consumed, to our harm. They get little chemically preserved and chemically coloured stuff. Heat, as I have shown, acts as a cancer poison. New arrivals at mental hospitals complain that the food is very plain and that the tea, porridge, etc., are never properly hot. We habitually take our tea, coffee, etc., at 150°, while the highest temperature of the hot bath is only 105°. Our stomach has no pain nerves, and, owing to chronic poisoning by chronic burning, thousands of people die every year from cancer of the oesophagus, of the stomach, etc. Mental hospital inmates are saved from that trouble.

The Government report mentioned, commenting on the low cancer death-rate in mental hospitals, and the fact that the cancer death-rate is stationary in these institutions while it is rapidly increasing among the people in general, urges cancer researchers to investigate the facts given, because they ought to throw light on the causation of cancer. The causation of that disease is no longer a mystery. It is plain to all who have eyes to see. No microscope is needed, and I am afraid that laboratory workers are overlooking the most important and the most significant facts. Cancer may never be curable, but it is certainly preventable. Prevention is better than cure.

J. Ellis Barker.

Albion Lodge, Fortis Green,
East Finchley, N. 2.

